# Pesticide exposure and the development of Parkinson disease: a systematic review of Brazilian studies

**DOI:** 10.1590/0102-311XEN011424

**Published:** 2025-04-11

**Authors:** Jean Rodrigo Santos, Marcello Calheiro Mendes, Kamila Gabrieli Dallabrida, Rithiele Gonçalves, Tuane Bazanella Sampaio

**Affiliations:** 1 Universidade Estadual do Centro-Oeste, Guarapuava, Brasil.; 2 Universidade Federal de Santa Maria, Santa Maria, Brasil.; 3 Hospital Universitário do Oeste do Paraná, Cascavel, Brasil.; 4 Universidade Estadual do Oeste do Paraná, Francisco Beltrão, Brasil.

**Keywords:** Agrochemicals, Neurodegenerative Diseases, Occupational Exposure, Environmental Exposure, Humans, Agroquímicos, Doenças Neurodegenerativas, Exposição Ocupacional, Exposição Ambiental, Humanos, Agroquímicos, Enfermedades Neurodegenerativas, Exposición Profesional, Exposición a Riesgos Ambientales, Humanos

## Abstract

Parkinson disease is the second most prevalent neurodegenerative disease globally. Parkinson disease etiology is not fully understood, it is believed to be a multifactorial disease. Pesticide exposure is highlighted among the factors. Thus, this study analyzed the relationship between pesticide exposure and the development of Parkinson disease in Brazil via a systematic review. The review was conducted following the PRISMA methodology and PICOS process, using the PubMed, Web of Science, and Virtual Health Library databases. Inclusion criteria were observational studies, conducted in humans, focusing on the Brazilian population, and investigating the relationship between pesticide exposure and Parkinson disease development. Studies quality was evaluated using the Hawker checklist. A total of 85 publications were identified for eligibility and 12 studies were included in the qualitative synthesis. Regarding study quality, two showed poor, nine moderate, and only one presented high quality. Moreover, 11 studies indicated an association between pesticide exposure and increased occurrence of Parkinson disease in Brazilian people. Additionally, such association was more prevalent in the presence of the following factors: (i) single-nucleotide polymorphism IVS1-7 A→G of *PINK1*; (ii) variations in the gene and protein expressions of the enzyme glutathione S-transferase; (iii) occupational exposure; (iv) living in a non-urban area; (v) low schooling level, and (iv) being male. This study is the first to infer, via the systematization of observational studies conducted with the Brazilian population, the association between pesticide exposure and the occurrence of Parkinson disease in the country, evidencing the necessity of efficient public policies.

## Resumo

A doença de Parkinson é a segunda doença neurodegenerativa mais prevalente globalmente. A etiologia da doença de Parkinson não é totalmente compreendida; acredita-se que seja uma doença multifatorial. A exposição a agrotóxicos é destacada entre os fatores. Assim, por meio de uma revisão sistemática, este estudo teve como objetivo analisar a relação entre a exposição a pesticidas e o desenvolvimento da doença de Parkinson no Brasil. A revisão foi conduzida de acordo com a metodologia PRISMA e o processo PICOS, utilizando as bases de dados PubMed, Web of Science e a Biblioteca Virtual em Saúde. Os critérios de inclusão foram estudos observacionais, realizados em humanos, com foco na população brasileira, investigando a relação entre a exposição a agrotóxicos e o desenvolvimento da doença de Parkinson. A qualidade dos estudos foi avaliada utilizando o *checklist* de Hawker. Um total de 85 publicações foi identificado para avaliação de elegibilidade, e 12 estudos foram incluídos na síntese qualitativa; dois foram classificados como de baixa qualidade, nove como de qualidade moderada e apenas um apresentou alta qualidade. Ao todo, 11 estudos indicaram uma associação entre a exposição a agrotóxicos e o aumento da ocorrência de doença de Parkinson na população brasileira. Além disso, essa associação foi mais prevalente na presença dos seguintes fatores: (i) polimorfismo de nucleotídeo único IVS1-7 A→G do gene *PINK1*, (ii) variações na expressão do gene e da proteína da enzima glutationa S-transferase, (iii) exposição ocupacional, (iv) residência em área não urbana, (v) baixo nível educacional e (vi) ser do sexo masculino. Para nosso conhecimento, este estudo é o primeiro a inferir, por meio da sistematização de estudos observacionais realizados com a população brasileira, a associação entre a exposição a agrotóxicos e a ocorrência da doença de Parkinson no país, evidenciando a necessidade de políticas públicas eficientes.

## Resumen

La enfermedad de Parkinson es la segunda enfermedad neurodegenerativa más prevalente a nivel mundial. Todavía no se conoce completamente la etiología de la enfermedad de Parkinson, y se la considera una enfermedad multifactorial. Entre los factores se destaca la exposición a pesticidas. A partir de una revisión sistemática, este estudio tuvo como objetivo analizar la relación entre la exposición a pesticidas y el desarrollo de la enfermedad de Parkinson en Brasil. La revisión se realizó de acuerdo con la metodología PRISMA y el proceso PICO mediante las bases de datos PubMed, Web of Science y Biblioteca Virtual en Salud. Los criterios de inclusión fueron estudios observacionales, en que se realizaron en humanos, que estuvieron centrados en la población brasileña y que investigaron la relación entre la exposición a pesticidas y el desarrollo de le enfermedad de Parkinson. La calidad de los estudios se evaluó mediante la lista de verificación de Hawker. Se identificaron un total de 85 publicaciones para evaluar la elegibilidad, y se incluyeron 12 estudios en la síntesis cualitativa; dos se consideraron de baja calidad, nueve de calidad moderada y solo uno de alta calidad. En total, 11 estudios indicaron una asociación entre la exposición a pesticidas y el aumento de la incidencia de la enfermedad de Parkinson en la población brasileña. Además, esta asociación fue más prevalente en presencia de los siguientes factores: (i) polimorfismo de un solo nucleótido IVS1-7 A→G del gen *PINK1*, (ii) variaciones en la expresión del gen y la proteína de la enzima glutatión S-transferasa, (iii) exposición ocupacional, (iv) residencia en un área no urbana, (v) bajo nivel educativo y (vi) ser del sexo masculino. Hasta el momento, este estudio es pionero en inferir, mediante la sistematización de estudios observacionales realizados con la población brasileña, la asociación entre la exposición a pesticidas y la ocurrencia de la enfermedad de Parkinson en el país, lo cual apunta a la necesidad de políticas públicas eficientes.

## Introduction

Parkinson disease was originally described over 200 years ago, in 1817, by James Parkinson. Although its pathophysiology remains uncertain, the degeneration of dopaminergic neurons in the substantia nigra pars compacta with consequent striatal dopamine decrease and the formation of Lewy bodies (protein aggregates containing α-synuclein) are neurochemical changes characteristic of Parkinson disease ^1^. Moreover, these changes culminate in the appearance of cardinal motor symptoms, such as bradykinesia, rigidity, postural instability, and rest tremor, which are essential for the clinical diagnosis of the disease ^2^.

Parkinson disease is the second most prevalent neurodegenerative disease, and aging is the main risk factor for its development ^3^. According to Ou et al. ^4^, a 155.5% increase in the prevalence of Parkinson disease was noticed from 1990 to 2019, reaching approximately 8.5 million people worldwide. That is, the number of people diagnosed with Parkinson disease has more than doubled, being associated with increased life expectancy, changes in lifestyle, and, possibly, environmental factors related to industrialization ^4^. In Brazil, due to the lack of notifications and epidemiological studies, most of the data on the incidence of Parkinson disease is estimated ^5^. Nevertheless, Dorsey et al. ^6^ estimated the global, regional, and country-specific prevalence of Parkinson disease from 1990 to 2016, ranking Brazil seventh among the Latin American countries.

The etiological factors of Parkinson disease are not fully understood. However, interactions between genetic and environmental factors are observed, classifying it as a multifactorial disease. Hereditary factors are present in approximately 10% of diagnosed cases of Parkinson disease, presenting early onset; while the other 90% of cases are categorized as idiopathic or sporadic Parkinson disease, occurring in older individuals and may be associated with exposure to environmental agents ^7^. In this sense, various factors are related to an increased risk of idiopathic Parkinson disease development, such as postmenopausal estrogen consumption, dairy products, methamphetamine, traumatic brain injury, melanoma, type 2 diabetes, living in rural areas, and exposure to pesticides ^7^ ^,^ ^8^.

Agriculture advancement elevated the use of pesticides and, consequently, the interest in its repercussions on human health. Although pesticides are beneficial to food production by protecting them against pests, contact with these substances generates acute poisoning and/or increased risk of developing chronic diseases by environmental and/or occupational exposure. In addition, such substances are toxic to other animal species besides agricultural pests, impacting various ecosystems ^9^. Based on this, numerous pesticides have been investigated for association with Parkinson disease. Of these, rotenone, paraquat, and maneb show toxicity mechanisms that have been most elucidated in pre-clinical studies ^9^ ^,^ ^10^ ^,^ ^11^ ^,^ ^12^ ^,^ ^13^.

Notably, from 1975 to 2008, Brazil was among the six largest consumers of pesticides in the world, taking the lead from 2008. Currently, Brazil is responsible for approximately 20% of the world’s pesticide consumption. Moreover, 381 of the 1,000 active ingredients of pesticides are approved for use in Brazil, being found in 2,400 different products ^14^. Therefore, due to (i) the elevated use of pesticides in Brazil; (ii) the high risk of developing chronic diseases associated with exposure to these substances; (iii) the participation of environmental factors in the etiology of Parkinson disease, and (iv) the evidence from basic research that some pesticides lead to the loss of dopaminergic neurons in the nigrostriatal area, we aimed to analyze, via a systematic review, the relationship between pesticide exposure and the development of Parkinson disease in Brazil.

## Materials and methods

### Study design and search strategy

This study is an exploratory-descriptive qualitative research, with the purpose of identifying, selecting, and synthesizing relevant evidence available, based on clear selection and eligibility criteria. In this sense, this systematic review followed the *Preferred Reporting Items for Systematic Reviews and Meta-Analyses* (PRISMA) guidelines and employed the PICOS process to elucidate the relationship between the events “development of Parkinson disease” and “pesticide exposure” ^15^. Box 1 provides an anagram of PICOS and its components.

**Box 1:** Components of the research question based on the PICOS process, focusing on pesticide exposure and the development of Parkinson disease in studies conducted in Brazil up to 2023.

ABBREVIATE	DESCRIPTION	COMPONENTS OF THE QUESTION
P	Population	Brazil
I	Intervention/Exposure	Pesticides
C	Comparison	Healthy vs. Parkinson disease
O	Outcome	Parkinson disease
S	Study design	Observational

Based on the components of the research question, three online databases were searched, namely PubMed, Web of Science, and Virtual Health Library, using *Medical Subject Headings* (MeSH) terminology. The following MeSH terms were used: *Brazil AND pesticides OR agrochemicals AND Parkinson’s disease*. Additionally, publications in any language up to the end of 2023 were considered. Moreover, the complete review protocol is registered in the PROSPERO international database (protocol n. CRD42023404607).

### Selection criteria and data extraction

All articles were independently reviewed for eligibility using the Rayyan software (https://www.rayyan.ai/). Once duplicates were deleted, two researchers (R.G. and T.B.S.) independently screened the titles, abstracts, and full texts to decide the studies’ eligibility. Discrepancies were resolved by consensus. The inclusion criteria for eligibility included: (i) observational primary studies; (ii) studies with humans; and (iii) studies carried out in the Brazilian population. The exclusion criteria comprised: (i) narrative/systematic/meta-analysis/editorial reviews or preprints publications; (ii) being carried out in other species that are not humans or in vitro/in silico systems; (iii) encompassing populations other than Brazilians. Studies were excluded if their outcomes did not address the development of Parkinson disease associated with pesticide exposure.

Data were extracted by two researchers (J.R.S. and M.C.M.) as follows: publication details (author names, title, and year of publication); study design (study type and number of individuals per group [Parkinson disease/non-Parkinson disease patient and/or exposure/non-exposure]); demographic data (Brazilian region, city, recruitment site); and main findings, with a focus on the putative association between Parkinson disease development in Brazilian people and pesticide exposure.

### Quality assessment

All eligible studies received a quality score using a checklist developed by Hawker et al. ^16^. This method was selected for its capacity to evaluate studies of various natures, providing a comprehensive and standardized overview of their quality. This instrument is based on nine domains: (1) title and abstract; (2) introduction and aims; (3) method and data; (4) sampling; (5) data analysis; (6) ethics and bias; (7) findings/results; (8) transferability/generalizability; and (9) implications and usefulness.

For each study, the nine domains were classified into one of four quality categories: very poor (1 point), when information was absent or inadequately described; poor (2 points), when information was lacking or descriptions were insufficient; fair (3 points), when information was present but with minor limitations or gaps; and good (4 points), when all necessary information was presented clearly and comprehensively ^16^. Then, the scores were summed for each study, and the overall quality score was ranked according to Dewa et al. ^17^, as follows: (A) high = 30-36 points; (B) medium = 24-29 points; and (C) low = 9-23 points. The quality assessment data of the included studies are presented as mean ± standard deviation of the mean of scores obtained independently by two researchers (R.G and T.B.S.).

## Results

**Figure 1:**
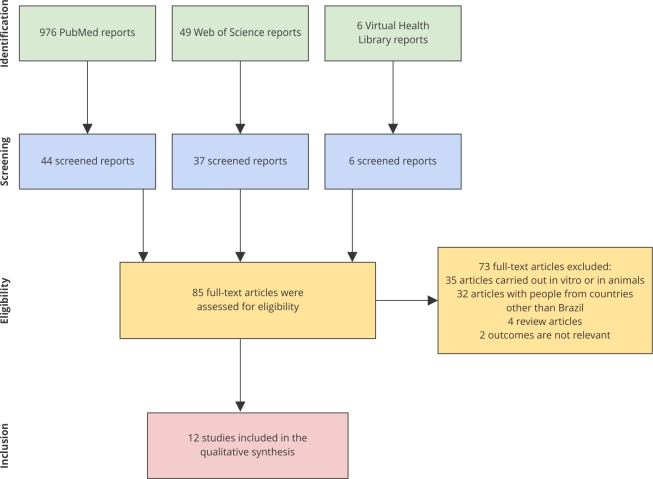
*Preferred Reporting Items for Systematic Review and Meta-Analysis* (PRISMA) flow diagram summarizing the selection process of studies included in the systematic review on the association between pesticide exposure and the development of Parkinson disease in Brazil, highlighting the identification, screening, eligibility, and inclusion phases, with data extracted from PubMed, Web of Science, and Virtual Health Library up to 2023.

According to the flowchart shown in Figure 1, the search in the databases resulted in the identification of 976 articles in PubMed, 49 articles in Web of Science, and 6 articles in Virtual Health Library. In PubMed, 44 reports were screened after the filter application: article type set for “case report”, “comparative study”, “multicenter study”, and “observational study”; species set for “humans”; and other set for “exclude preprints”. In turn, 37 reports remained after the document types filter (including “article”, “data paper”, “early access”, “letter”, and “proceeding paper”) in Web of Science. No study was removed from the initial search in the Virtual Health Library.

Then, 87 scientific studies linked to the events “development of Parkinson disease” and “pesticide exposure” were selected and entered into the Rayyan software. Of these, two studies were duplicated, thus 85 full-text articles remained for eligibility analysis. A total of 73 scientific articles were discarded for being research carried out with experimental animals, cells, or in silico analysis (n = 35), having as target population people from countries other than Brazil (n = 32), being article reviews (n = 4), or investigating an outcome not relevant for this systemic review (n = 2); that is, for not corresponding to the pre-established eligibility criteria (Figure 1).

Therefore, 12 articles were included in the qualitative synthesis, published from 1988 to 2023. In summary, the publications included in the qualitative synthesis are primary studies containing original clinical observational data, which among their findings, elucidate the association between the development of Parkinson disease in pesticide-exposed Brazilians, both via occupational and environmental exposures. Box 2 displays the characterization of the studies regarding the design, sampling with and/or without Parkinson disease, region of the country, and the main findings of the research. Overall, based on the analyzed studies, we suggest an association between pesticide exposure and the development of Parkinson disease in Brazil.

**Box 2:** Data extraction from eligible studies published from 1988 to 2023 investigating the relationship between pesticide exposure and the development of Parkinson disease in the Brazilian population.

STUDY	STUDY DESIGN	SUBJECTS	COUNTRY REGION	MAIN FINDINGS
Ferraz et al. ^18^ (1988)	Case-control	15 rural workers exposed to maneb and 19 controls	Southeast	There was a higher prevalence of cogwheel phenomenon, fatigue, nervousness, headache, memory impairments, and sleepiness in the maneb-exposed subjects
Werneck & Alvarenga ^25^ (1999)	Case-control	92 Parkinson disease patients and 110 controls	Southeast	No significant association between pesticide exposure and Parkinson disease was found
Barbosa et al. ^19^ (2001)	Case report	A man accidentally intoxicated himself with glyphosate	Southeast	Parkinsonian syndrome after one month and bilateral hyperintense signal in the globus pallidus and substantia nigra two years after intoxication. Levodopa/benserazide treatment provided clinical benefit
Godeiro Jr. et al. ^22^ (2010)	Cross-sectional	48 Parkinson disease patients and 61 controls	Southeast	The *PINK1* SNP IVS1-7 A→G associated with exposure to environmental factors decreased the age for onset of early-onset Parkinson disease
Pinhel et al. ^24^ (2013)	Case-control	254 Parkinson disease patients and 169 controls	Southeast	Parkinson disease was associated with pesticide exposure, with an increased effect by *GSTT1*/*GSTM1* null genotypes
Longo et al. ^23^ (2013)	Case-control	154 Parkinson disease patients and 158 controls	Southeast	Heterozygous polymorphism for *GSTP1*-Alw26I is prevalent in Parkinson disease patients exposed to pesticides and the homozygous genotype is found only in familial Parkinson disease. The association between Parkinson disease and pesticide exposure is enhanced by the I/V genotype of *GSTP1*-Alw26I
Vasconcellos et al. ^33^ (2019); Vasconcellos et al. ^32^ (2020)	Cross-sectional	32 Parkinson disease patients	South	They find an association between the time of exposure to pesticides, both via direct and indirect contact, and the development of Parkinson disease
Schneider Medeiros et al. ^20^ (2020)	Cohort	233 Parkinson disease patients: 150 (pesticide exposure form) and 126 (socioeconomic questionnaire)	South	The mortality of Parkinson disease patients was found to increase with occupational exposure to pesticides
Silvestre et al. ^31^ (2020)	Case-control	88 Parkinson disease patients and 264 controls	Central-West	The factors being male, over 60 years old, having low schooling level and family history of Parkinson disease increase the probability of developing Parkinson disease. Direct management of pesticides and occupational exposure are the situations that most increase the risk of developing Parkinson disease
Santos et al. ^35^ (2021)	Ecological	52 urban and 500 non-urban microregions	Brazil	They find a correlation between the Parkinson disease hospitalization rate in non-urban microregions and pesticide expenditures, reaching the Parkinson disease hospitalization rate ratio of 5.90 in microregions of higher pesticide use
Moura et al. ^34^ (2023)	Cross-sectional	562 Parkinson disease patients and 374 controls	North, South, and Southeast	Household pesticide exposure for more than 30 days per year at any time during their lifetime implies two times more risk of developing Parkinson disease, without impacting in age at Parkinson disease onset

However, for a conclusion, it was necessary to evaluate the methodological rigor of the studies. In this way, two studies showed poor quality, nine were scored as moderate, and only one reached a high-quality score (Table 1). Study design, sampling, generalizability, and implications were some weaknesses found in moderate-quality publications. Ferraz et al. ^18^ and Barbosa et al. ^19^ studies also presented the weaknesses observed in moderate-quality papers added to unclear ethical procedures, resulting in a poor-quality score. Only the retrospective cohort study by Schneider Medeiros et al. ^20^ reached a high-quality score. This high-quality paper provided clear and coherent research aims, plausible rationale for the study, detailed characterization of the sample and research design, concise information about ethical considerations and possible biases, rigorous explanations of the data analysis to enable replicability, and adequate literature reviews to contextualize the study.

**Table 1:** Quality assessment employing the Hawker checklist, including score and final classification, of eligible studies published from 1988 to 2023 investigating the relationship between pesticide exposure and the development of Parkinson disease in the Brazilian population.

Study	Score (mean ± SD)	Quality
Ferraz et al. ^18^ (1988)	20.5 ± 0.7	Poor
Werneck & Alvarenga ^25^ (1999)	26.0 ± 0.0	Moderate
Barbosa et al. ^19^ (2001)	19.5 ± 2.1	Poor
Godeiro Jr. et al. ^22^ (2010)	27.5 ± 0.7	Moderate
Pinhel et al. ^24^ (2013)	29.0 ± 0.0	Moderate
Longo et al. ^23^ (2013)	28.0 ± 1.4	Moderate
Vasconcellos et al. ^33^ (2019)	25.0 ± 0.0	Moderate
Vasconcellos et al. ^32^ (2020)	28.5 ± 0.7	Moderate
Schneider Medeiros et al. ^20^ (2020)	31.5 ± 0.7	High
Silvestre et al. ^31^ (2020)	28.5 ± 0.7	Moderate
Santos et al. ^35^ (2021)	26.5 ± 2.1	Moderate
Moura et al. ^34^ (2023)	27.0 ± 0.0	Moderate

SD: standard deviation.

## Discussion

Since Parkinson disease etiology presents both environmental and genetic factors, it is understood as a multifactorial neurodegenerative disease. Furthermore, Parkinson disease pathogenesis has been divided into three stages, according to the influence of these factors: (i) triggers or initiators; (ii) facilitators; and (iii) aggravators. During the trigger stage, viral infections or environmental toxins, i.e., pesticides, cause neural damage in central and/or peripheral tissues. However, only triggers are insufficient, requiring facilitators such as cellular senescence and genetic characteristics to lead to Parkinson disease development. Once manifested, aggravators, such as α-synuclein aggregates, stimulate the neurodegeneration progression ^21^.

In this context, four of the 12 publications included in this review considered the multifactorial etiology of Parkinson disease ^22^ ^,^ ^23^ ^,^ ^24^ ^,^ ^25^. Among them, only the study by Werneck & Alvarenga ^25^ found no association between pesticide exposure and Parkinson disease development but suggested drug use and family history, which act as putative trigger and facilitator, respectively, as risk factors for Parkinson disease. However, it is important to highlight the study site to understand the absence of pesticide correlation. The subjects were recruited in 1996 and 1997 from the Neurology Department of a central hospital in Rio de Janeiro, an irrelevant area for agricultural productivity. Selected individuals for studying pesticide exposure as a risk factor totaled six Parkinson disease patients (6.36%) and three controls (2.72%). Of note, four of nine individuals reported occupational use of pesticide during their time living in rural areas, and all had Parkinson disease ^25^.

On the other hand, the studies by Godeiro Jr. et al. ^22^, Longo et al. ^23^, and Pinhel et al. ^24^ corroborate the hypothesis that environmental risk factors, including pesticides, could be triggers and changes in gene or protein expression could act as facilitators for the development of Parkinson disease. Godeiro Jr. et al. ^22^ evaluated whether the presence of single nucleotide polymorphisms (SNPs) in the *PINK1* gene plus exposure to environmental risk factors impacts the clinical presentation of early-onset Parkinson disease. *PINK1* protein has a neuroprotective role by inhibiting proteasome formation and regulating the degradation of damaged mitochondria ^26^. In this way, 48 patients with early-onset Parkinson disease and 61 control subjects were recruited from the ambulatory of the Federal University of São Paulo and the Hospital Israelita Albert Einstein from 2004 to 2008. The *PINK1* SNP IVS1-7 A→G was mostly found in patients with early-onset Parkinson disease. Additionally, the association of *PINK1* SNP IVS1-7 A→G with exposure to environmental factors reduced the age for onset of Parkinson disease, corroborating that genetic and environmental factors may act together in Parkinson disease pathogenesis ^22^.

The enzyme glutathione S-transferase (GST) has also been the target of investigation in Parkinson disease patients exposed to pesticides ^23^ ^,^ ^24^. GST, as well as its gene variants *GSTM1*, *GSTT1*, and *GSTP1*, show an antioxidant function, acting in the neutralization of oxidative stress products and xenobiotics ^27^. Thus, a decrease in its activity impacts the detoxification capacity of cells, especially in individuals exposed to pesticides. Pinhel et al. ^24^ reported the involvement of null genotypes for the *GSTM1* and *GSTT1* variants, catalyzed by the environment, as possible facilitators for Parkinson disease development. Indeed, they demonstrated - through a case-control study containing 254 Parkinson disease patients and 169 control individuals from the Movement Disorders Ward of the Base Hospital of the Faculty of Medicine in São José do Rio Preto - a higher frequency of *GSTM1* and *GSTT1* nullity when the patient had contact with pesticides. Since *GSTM1* and *GSTT1* act in the metabolism of xenobiotics, oxidative stress markers were altered in Parkinson disease patients exposed to pesticides ^24^. Therefore, oxidative stress may be an aggravator in the neurodegenerative progression.

Similarly, the frequency of the *GSTP1*-Alw26I polymorphism in Parkinson disease patients exposed to toxins was evaluated ^23^. Of note, the genotypic distribution of the *GSTPq*-Alw26I polymorphism in the Brazilian population is similar to that of the general population ^28^ ^,^ ^29^ ^,^ ^30^. Thus, through a case-control study with 154 Parkinson disease patients and 158 control subjects, Longo et al. ^23^ demonstrated that the heterozygous polymorphism for *GSTPq*-Alw26I is prevalent in Parkinson disease patients exposed to pesticides. On the other hand, the homozygous genotype was associated only with familial Parkinson disease patients. Moreover, pesticide exposure was associated with the Parkinson disease development, which can be potentiated by the I/V genotype of *GSTP1*-Alw26I. Occupational exposure to pesticides also impacted a larger male and older adult population ^23^.

Corroborating these findings, the other studies included in this systematic review also found an association between pesticide exposure and the occurrence of Parkinson disease in individuals from the Southeast, South, North, and Central-West of Brazil ^18^ ^,^ ^20^ ^,^ ^31^ ^,^ ^32^ ^,^ ^33^ ^,^ ^34^ ^,^ ^35^. Ferraz et al. ^18^ conducted a pioneering investigation on the signs and symptoms shown by maneb-exposed rural workers for more than six months. At that date, there was a single published report of central nervous system changes due to acute exposure to maneb ^36^ and they found two patients with Parkinsonian syndrome that was associated with previous occupational maneb exposure ^18^. Besides the reduced number of research subjects (15 maneb-exposed rural workers with 19 rural worker controls from a small town in the Southeast region of Brazil), they showed a higher prevalence of cogwheel phenomenon (a type of rigidity often seen in early Parkinson disease), fatigue, nervousness, headache, memory impairments, and sleepiness in maneb-exposed individuals, suggesting that maneb exposure may be an environmental factor for Parkinson disease ^18^.

Maneb, a dithiocarbamate fungicide containing manganese (Mn), is one of the mancozeb’s components, the second most commonly used pesticide in Brazil in 2023 ^37^. In this sense, both mancozeb and maneb inhibit the mitochondrial respiratory chain, leading to increased reactive oxygen species, motor impairments, and degeneration of nigrostriatal dopaminergic neurons in preclinical studies ^13^ ^,^ ^38^ ^,^ ^39^. Although molecular mechanisms remain unclear, recent findings suggest that maneb disrupts the neurotransmitter synthesis and stimulates the asparagine endopeptidase - a lysosome-associated cysteine protease that cleaves the α-synuclein - in mice, resulting in Parkinson disease-like phenotype ^38^.

Parkinsonian syndrome after exposure to another pesticide has also been documented in a case report in Brazil. A 54-year-old man, without a familial history of Parkinson disease, accidentally sprayed himself with glyphosate ^19^, the most commercialized herbicide in Brazil ^37^ and worldwide. Initially, one month after the acute intoxication, he presented slowness and rigidity in all four limbs, progressing to resting tremors in the left arm and hand and short-term memory deficit one year later. Levodopa/benserazide treatment began two years after the accident and the clinical benefit confirmed the Parkinsonian syndrome, as well as the hyperintense bilateral lesions in the substantia nigra and globus pallidus in the magnetic resonance imaging and clinical signs (global akinesia, cogwheel phenomenon, postural instability, and resting tremor in left limb) ^19^. The mechanism of action that underlines glyphosate-induced neurotoxicity is uncertain and dopaminergic changes seem to be transient ^40^. Notably, in adult rats, dopamine depletion was observed in nigrostriatal and mesocorticolimbic pathways, along with reduced D1 binding after glyphosate administration ^41^, as well as oxidative stress, neuroinflammation, and behavioral deficits ^40^.

In this sense, glyphosate along with hexachlorobenzene and paraquat were the pesticides most often mentioned by the interviewees from Western Paraná University Hospital from 2012 to 2017. During this period, 507 attendances were performed, of which 48 presented Parkinson disease diagnosis and 32 agreed to be interviewed ^32^ ^,^ ^33^. They found a correlation between the risk of developing Parkinson disease and pesticide exposure when patients showed lower schooling levels ^32^. In addition, most of the patients lived from 11 to 30 years in rural areas and about 75% reported a history of direct contact with pesticides. Only 25% of these reported the use of personal protective equipment, such as boots and masks. The most cited form of pesticide application was with costal spray. Moreover, 75% of patients reported a time greater than 20 years between direct exposure to pesticides and the onset of Parkinson disease symptoms ^32^ ^,^ ^33^.

Although the number of subjects and study design are important limitations of these studies ^18^ ^,^ ^19^ ^,^ ^32^ ^,^ ^33^, they paved the way for more robust research. Schneider Medeiros et al. ^20^ demonstrated, in a retrospective cohort of 233 Parkinson disease patients from Porto Alegre Clinical Hospital (of these, 150 respondents of a pesticide exposure form and 126 respondents of a socioeconomic questionnaire), that occupational exposure to pesticides doubled the mortality of patients with Parkinson disease when compared to non-exposed patients. A higher mortality rate associated with occupational exposure to pesticides was also observed in patients with low income and low schooling level ^20^.

A case-control study carried out at the Mato Grosso State General University Hospital, including 88 Parkinson disease patients and 264 control individuals, also showed that direct handling of pesticides in the workplace increased the probability of developing Parkinson disease by more than three times (odds ratio [OR] = 3.78; 95% confidence interval [95%CI]: 1.92-7.45) ^31^. Interestingly, the risk of developing Parkinson disease due to pesticide exposure was similar to those with a family history of the disease (OR = 3.42; 95%CI: 1.61-7.28). Moreover, Silvestre et al. ^31^ found that being male, over 60 years of age, and having low schooling level increases the probability of developing Parkinson disease. Nevertheless, occupational exposure to pesticides shows the greatest risk. Tobacco and alcohol consumption showed protective factors for the occurrence of Parkinson disease ^31^.

Low schooling level is a silent and chronic issue in developing countries ^20^ ^,^ ^32^. In this study, we described a consistent association between poor schooling and increased risk of developing Parkinson disease in people exposed to pesticides, suggesting that the inability to read the toxicity warnings makes them susceptible to the risks of pesticides. Poverty is related to premature death due to neurodegenerative diseases worldwide ^42^. Controversially, Silvestre et al. ^31^ showed that family income below one minimum wage could be a protective factor for Parkinson disease. According to them, low income is related to decreased occupational or environmental exposure to pesticides because these individuals do not depend on agribusiness ^31^.

Notably, among the pesticides cited, hexachlorobenzene and paraquat have been banned in Brazil since 1985 and 2017 ^43^, respectively, evidencing the lack of knowledge and awareness regarding the health dangers. On the other hand, the herbicide diquat dibromide is allowed in Brazil and was the tenth most commercialized pesticide in 2023 ^37^. Diquat and paraquat are bipyridyl compounds structurally similar to each other and to 1-methyl-4-phenyl-pyridinium, the active metabolite of 1-methyl-4-phenyl-1,2,3,6-tetrahydropyridine (MPTP) - a neurotoxin that can cause Parkinsonism in humans and is largely used to model Parkinson disease ^44^. Diquat was shown to cause damages to the mitochondrial bioenergetics and locomotor functions in zebrafish ^45^. Interestingly, diquat-induced cell death seems mainly linked to the necrosis process and reactive oxygen species are formed independently of mitochondria ^46^ ^,^ ^47^. Additionally, the annual report on the production, import, export, and sales of pesticides in Brazil (2023) highlighted that, alongside the pesticides previously mentioned, 2,4-dichlorophenoxyacetic acid (2,4-D), acephate, chlorothalonil, atrazine, S-metolachlor, glufosinate-ammonium salt, and malathion were also among the ten most commercially sold pesticides ^37^. Of note, 2,4-D, atrazine, and malathion have evidence linking their mechanisms of action to the Parkinson disease pathophysiology ^47^ ^,^ ^48^ ^,^ ^49^.

Brazil has led the pesticide use in the world, and Santos et al. ^35^ conducted a study to understand the association between pesticide expenditure and Parkinson disease hospitalization rates in Brazilian microregions, finding significant correlations both in urban and non-urban microregions. Nevertheless, the highest pesticide expenditures are associated with non-urban microregions, suggesting that rural living may experience higher environmental or occupational exposure. Microregions of higher pesticide consumption displayed Parkinson disease hospitalization rates up to 5.90, showing associations with pesticide expenditure even in the lower age groups ^35^.

Interestingly, household exposure to pesticides also has been investigated in the North, South, and Southeast of Brazil. Household exposure was defined as the use of chemical substances to kill insects, mold, weeds, or other pests in/or around the house/apartment where they lived during their lifetime. Risk of developing Parkinson disease doubled in individuals exposed to household pesticides for more than 30 days per year at any time during their lifetime ^34^.

The scarcity of studies on the relationship between pesticide exposure and the occurrence of Parkinson disease in the Brazilian population is notable, especially when compared to the scientific output from other countries. Despite Brazil being the world’s largest consumer of pesticides, followed by the United States, the amount of research on this topic in Brazil is disproportionate. A meta-analysis of 46 studies published from 1950 to November 2010 revealed that only one study was conducted in Brazil, compared to 16 in the United States ^50^. Similarly, a meta-analysis covering research from January 1947 to August 2010 included only one Brazilian study among the 44 analyzed, while 13 were from the United States ^51^. Moreover, another review of observational case-control studies published up to April 2016 highlighted this disparity: of the 64 studies reviewed, only one was from Brazil, and 28 were conducted in the United States ^52^.

Besides the evident scarcity of studies focused on the Brazilian population, these data demonstrate the absence of robust studies investigating the relationship between Parkinson disease development and pesticide exposure. This is particularly alarming given that Brazil is the largest consumer of pesticides in the world. Among the included studies, only one was a retrospective cohort study, and prospective cohorts are considered the best study design to infer the causality between two events. Case-control and cross-sectional studies are methodologically fragile due to bias susceptibility. However, cross-sectional studies evaluate exposure and effect simultaneously at a single point in time, avoiding the inference of causality. On the other hand, well-designed case-control studies with an adequate number of subjects can infer potential causal associations between disease occurrence and exposure ^53^, as analyzed in this review. Notably, of the five case-control studies included in the qualitative synthesis, only one shows poor quality.

Nevertheless, the absence of robust data on the prevalence of Parkinson disease in Brazil underscores the lack of knowledge about the national situation. Such a research gap may result in an underestimation of the risks and lead to insufficiently informed public policies to mitigate the impacts of pesticide exposure on the health of the Brazilian population. Moreover, in addition to the use and exposure to illegal products, there is a lack of knowledge and awareness regarding the health dangers. This situation highlights the need to establish surveillance and health education initiatives to prevent exposure to illegal substances and promote proper pesticide handling practices. 

## Conclusions

In summary, this study investigated and inferred, for the first time, via the systematization of clinical studies conducted with the Brazilian population, the association between exposure to pesticides and the occurrence of Parkinson disease in Brazil, especially in rural areas with predominant agricultural activities. Considering the multifactorial etiology of Parkinson disease, studies conducted with the population in the Southeast of the country showed that the combination of the *PINK1* SNP IVS1-7 A→G polymorphism with exposure to pesticides reduces the age of onset of the disease. It was also observed that the association between Parkinson disease and pesticide exposure is intensified in the presence of null genotypes for the *GSTM1* and *GSTT1* genes. Similarly, the heterozygous polymorphism for *GSTP1*-Alw26I was prevalent in Parkinson disease patients exposed to pesticides. Thus, genetic predispositions, such as variations in the *PINK1* and *GST* genes, seem to amplify the effects of pesticide exposure, resulting in earlier onset and progression of Parkinson disease. In turn, studies conducted with the population of the South and Central-West of Brazil revealed that the duration of exposure to pesticides is associated with the development of Parkinson disease, with occupational exposure being the highest risk factor for Parkinson disease development and increasing mortality in Parkinson disease patients. Moreover, studies covering different Brazilian regions corroborate a positive correlation between pesticide expenditures and Parkinson disease hospitalization rate, and reveal that household exposure to pesticides constitutes a risk factor for developing Parkinson disease.

Thus, these data show that pesticide exposure, including glyphosate, paraquat, and maneb, can be associated with Parkinson disease development. Despite the strong associations identified, there are significant gaps in current research. No prospective cohort study have investigated the relationship between Parkinson disease development and pesticide exposure in Brazil, and the effects of less common pesticides are still poorly understood. Also, the North and Northeast regions of Brazil remain underrepresented in studies. Future research should aim to fill these gaps and provide a more comprehensive understanding of Parkinson disease across Brazil’s diverse agricultural scenarios. Expanding research efforts to include a broader range of pesticides, genetic factors, and underrepresented regions is essential to develop effective strategies to mitigate the risk of Parkinson disease associated with pesticide exposure in the country. In this context, it is crucial to propose public health policies to reduce pesticide exposure, including stricter regulations on pesticide use, improved safety protocols for agricultural workers, and comprehensive educational campaigns to raise awareness of the associated risks of pesticide exposure. In addition, public health initiatives should focus on monitoring pesticide levels in agricultural areas and implementing screening programs for early detection of Parkinson disease symptoms among rural workers.
